# The Therapeutic Administration of Iron

**Published:** 1897-12

**Authors:** L. A. Felder

**Affiliations:** Atlanta, Ga.


### THE THERAPEUTIC ADMINISTRATION OF IRON.

By L. A. FELDER, M.D.,

Atlanta, Ga.

While much space has been recently given by the journals to this important therapeutic agent, and much valuable information has been thrown on the subject by the practical physicians of the day, the manufacturing chemists have also labored hard to place before the medical profession iron in its purest and most delicate form for ready assimilation into the system.

Dating back to the stress paid to the proper administration of iron by Professor Roberts Bartholow, and to the rapid strides made by the chemists of the day, there is absolutely no excuse for hesitancy in the administration of iron for fear of deranging the digestion, etc. We no longer have to administer iron in such crude form and massive doses that will and has caused many unpleasant and disagreeable conditions and effects. Not longer than two months ago I was called in consultation with one of Southwest Georgia's prominent physicians. The case in question was one, for six months past, slight fever at intervals, evidently due to malaria. Patient could not take quinine. Arsenic had been given, still slight fever; patient anemic and quite nervous. We agreed to double the dose of arsenic and in addition give one of the latest preparations of iron. Physician in charge at first stated to me that his experience with iron in that climate was a complete failure. However, he agreed to try it in this case, and the result was, in a few weeks he was satisfied that iron administered in this case was *the* treatment, as our patient rapidly improved and regained healthy color and solid flesh as result of treatment.

For the past twelve months I have clearly demonstrated to my complete satisfaction that iron, as we now have several excellent pharmaceutical preparations of, prepared by our best manufacturing chemists for ready absorption into the system, will do more good administered to anemic and debilitated subjects, to restore vigor and a truly healthy condition of the impoverished blood, than any known remedial agent that we now possess. Bartholow, in his excellent work on Materia Medica, dating a number of years back, clearly demonstrates the most important step towards the assimilation of iron into the system, and the chemists have endeavored to combine it with those agents that favor the absorption of same. The administration of iron is indicated in cases of sluggish circulation of the skin, particularly of the face, as shown by a muddy and oily condition of the skin, unpleasant formation of blotches and pimples. We undoubtedly have in those cases deficient amount of healthy red-blood corpuscles. An evidence of the need of iron in such cases: paleness of the lip borders and of the conjunction of the eyelids. Ferratin has been used in quite a number of these cases with gratifying results; a prompt restoration of the skin to healthy color and removal of pimples, etc., with evidence of general improvement in health of patient in every instance. Ferratin, given in such circumstances, might well be called a beautifier of the complexion, as well as a restorer of health and vigor in many forms of anemic and debilitated conditions. In a number of such cases my experience with Ferratin has taught me that we may safely rely upon it as one of our best modes of administering iron.

---

"THE trail of the microbe is over them all," may well be written of every appliance that is to be found in the druggist's shop, or in even the best cared for sick room, says the *Independent*. The latest offender is the common rubber dropper for eye lotions. A dropper soon after being used becomes covered with a layer of bacteria, and they become coated with an insoluble white flourlike film. The lotion itself has probably been carefully sterilized, but the instant it touches the bulb the film comes off, and the insoluble particles are diffused through the liquid. When one reflects that the amount of contaminated liquid that Tyndall took up on a thread of spun glass was enough to infest a large test tube of sterilized material, and fill it with swarming microbes, we appreciate the force of thoroughly cleansing the dropper.—*Scientific American.*

